# Endocrine pancreatic development: impact of obesity and diet

**DOI:** 10.3389/fphys.2013.00170

**Published:** 2013-07-18

**Authors:** Jacqueline F. O'Dowd, Claire J. Stocker

**Affiliations:** Metabolic Diseases Group, Clore Laboratory, University of BuckinghamBuckingham, UK

**Keywords:** maternal over-nutrition, obesity, developmental programming, pancreas, transcription factors

## Abstract

During embryonic development, multipotent endodermal cells differentiate to form the pancreas. Islet cell clusters arising from the pancreatic bud form the acini tissue and exocrine ducts whilst pancreatic islets form around the edges of the clusters. The successive steps of islet differentiation are controlled by a complex network of transcription factors and signals that influence cell differentiation, growth and lineage. A Westernized lifestyle has led to an increased consumption of a high saturated fat diet, and an increase in maternal obesity. The developing fetus is highly sensitive to the intrauterine environment, therefore any alteration in maternal nutrition during gestation and lactation which affects the *in-utero* environment during the key developmental phases of the pancreas may change the factors controlling β-cell development and β-cell mass. Whilst the molecular mechanisms behind the adaptive programming of β-cells are still poorly understood it is established that changes arising from maternal obesity and/or over-nutrition may affect the ability to maintain fetal β-cell mass resulting in an increased risk of type 2 diabetes in adulthood.

## Introduction

The pancreas is composed of both exocrine and endocrine tissue. The exocrine gland secretes digestive enzymes while the endocrine gland, which consists of the islets of Langerhans, secretes hormones into the bloodstream. Each individual islet is composed of a number of endocrine cells specializing in the secretion of specific hormones (Bonner-Weir and Orci, 1982). There are four main endocrine cells contained within an islet: insulin producing β-cells; α-cells, which produce glucagon; δ-cells, which produce somatostatin and PP-cells, which secrete pancreatic polypeptide.

The development of the pancreas is a complex process. During embryogenesis the pancreas is derived from the gut and a cascade of signaling processes acts on precursor cells to determine their cell fate generating both exocrine and endocrine cells prior to birth. In rodents, β-cell neogenesis, the generation of new β-cells (Paris et al., 2004) continues throughout neonatal life, but ceases shortly after weaning (Deltour et al., 1991). Any environmental stress or stimulus during these critical periods of early development can permanently alter endocrine mass and β-cell function resulting in long-term health consequences in the developing infant (McMillen and Robinson, 2005).

**Figure 1:**
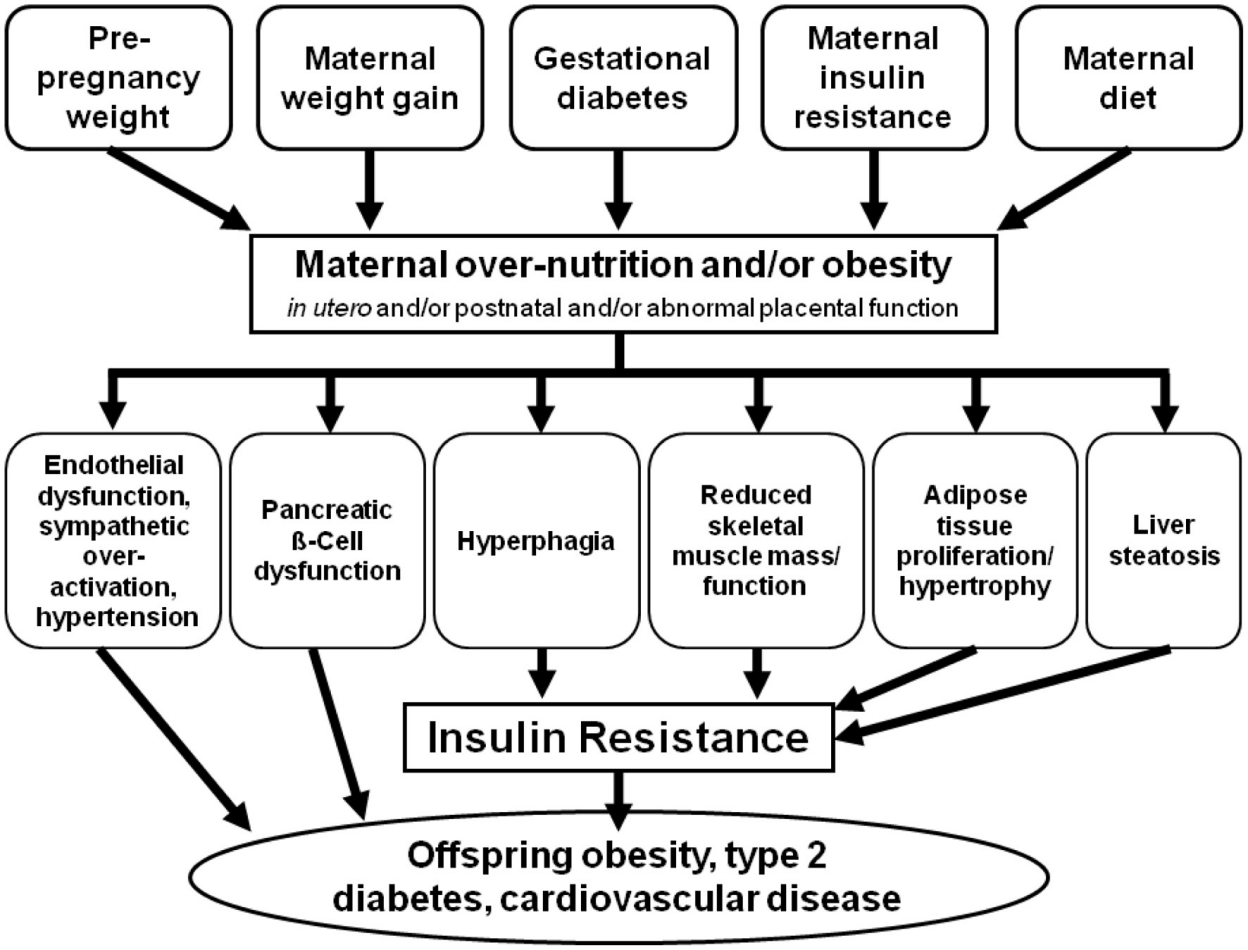
**Common mechanistic pathways of developmental programming as a result of maternal over-nutrition and obesity**.

Over recent decades there has been a rapid rise in metabolic disorders throughout the world. A Westernized lifestyle and societal habits have contributed to the obesity and type 2 diabetes epidemics. The WHO estimates that in 2008 there were more than 1.4 billion overweight adults worldwide, 500 million of which are obese (WHO, 2013). As the number of obese individuals increase, and in particular in women of child-bearing age, so does the rate of obese pregnancies. There is now increasing evidence that the early developmental environment of an infant can play a pivotal role in the “programming” of an adverse physiological phenotype in later life. Clinical evidence highlights that maternal over-nutrition and/or obesity presents not only adverse effects on maternal health, but also persistent and deleterious effects in the developing child (Nelson et al., 2010; Alfaradhi and Ozanne, 2011; Poston, 2012; Rkhzay-Jaf et al., 2012) (Figure 1).

In clinical studies it is harder to dissect out the effects of maternal obesity from those of an obesogenic diet, however, this can be addressed in animal models. Animal models provide essential insight into the underlying cellular and molecular mechanisms that contribute to this adverse phenotype. The majority of research has focused on sheep and rodents (Li et al., 2011). While many developmental events occur postnatally in rodents, the short gestation period, development of the metabolic syndrome within months, cost effectiveness and longitudinal nature of these studies, means that rodents are the most commonly used models (Alfaradhi and Ozanne, 2011). Sheep and non-human primates do offer advantages because of the similarities of their developmental patterns to humans; however, long gestation periods and high costs make them less available. The information gained from these animal studies not only aids our understanding of the programming signals related to maternal and paternal over-nutrition but may enable the improved healthcare for both mother and infant. This review will firstly describe the development of the pancreatic β-cell and will then examine the impact of developmental programming as a result of maternal over-nutrition on these underlying mechanisms and the resulting phenotype of the offspring.

## Development of the endocrine pancreas

### Embryonic development of the pancreas

Gastrulation is a key stage in early embryonic development. Inward migration of cells at or near the surface of the embryo reorganizes the single layered blastula into a three layered structure composed of the ectoderm, mesoderm and endoderm germ layers. Each layer gives rise to specific tissues and organs in the developing embryo. The pancreas and other gastrointestinal organs are derived from the endoderm.

**Figure 2:**
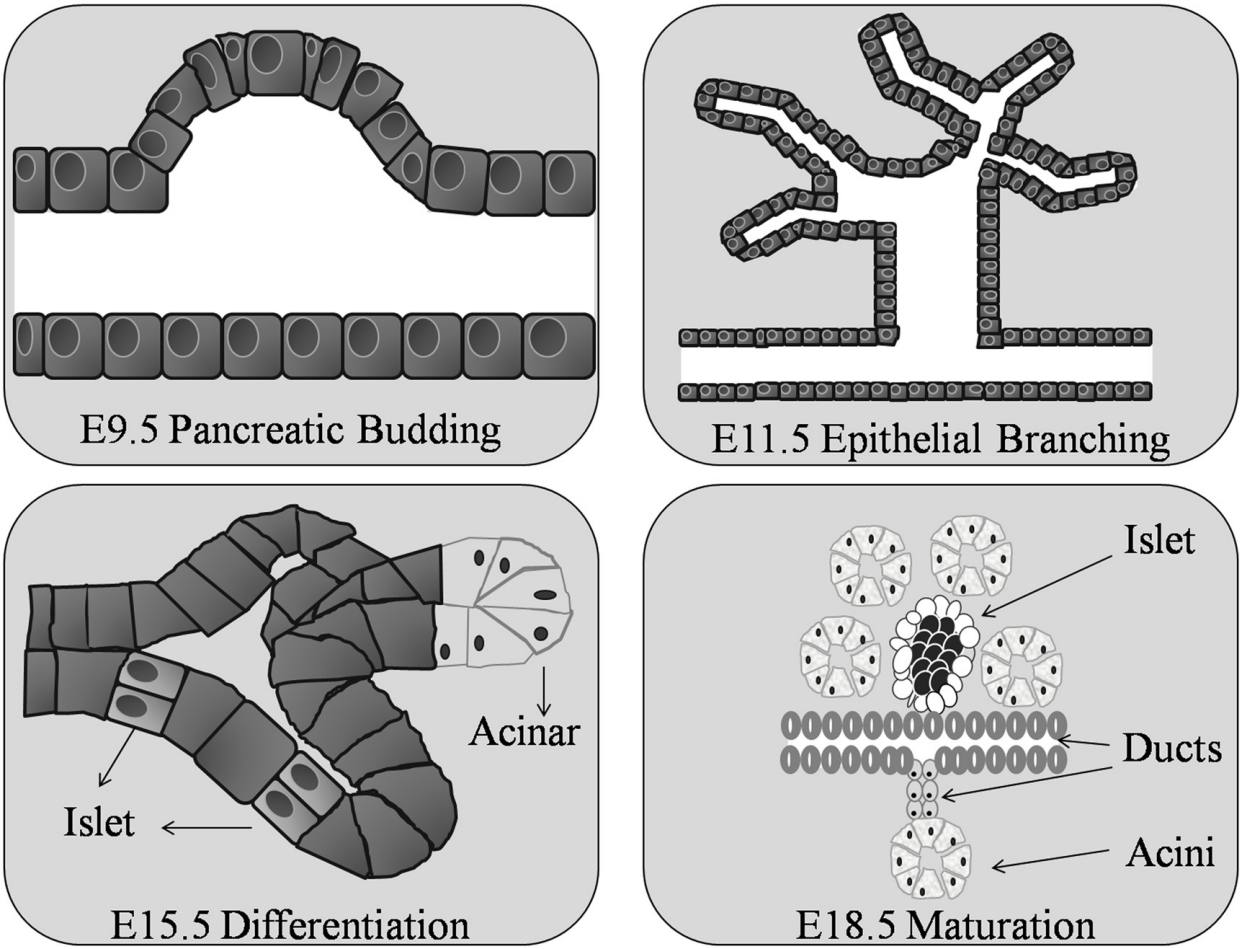
**Organogenesis of the murine pancreas.** The pancreatic bud begins around day 9 of gestation (E9.5) in the mouse embryo. At E11.5 the pancreatic ductal epithelium grows and branches into the surrounding mesenchyme. The epithelial cells differentiate into exocrine and endocrine cells at E15.5. Ductal, acinar, and islet cells are clearly found by E18.5.

The pancreas is formed in the uterus from the fusion of two separate pancreatic ducts, the dorsal and ventral. The dorsal pancreatic bud first appears at approximately embryonic day 9.5 (E9.5) in the mouse (Figure 2) [day 25 of gestation in humans (Piper et al., 2002)], as a diverticulum from the dorsal aspect in the primitive gut endoderm, in the area that will become the duodenum, a short distance above the hepatic diverticulum (Wessells and Cohen, 1967). Shortly after, the ventral pancreatic bud appears as a diverticulum from the primitive bile duct. By E11.5 in the mouse the two buds grow rapidly sending finger-like epithelial protrusions into the surrounding mesenchyme leading to the formation of highly branched structures (Zhou et al., 2007; Figure 2).

The dorsal duct grows upward and backward into the dorsal mesogastrium, forms a part of the head and uncinate process and the whole of the body and tail of the pancreas; while the ventral duct forms the remainder of the head and uncinate process of the pancreas (Gittes, 2009). The dorsal duct drains the major portion of the embryonic pancreas into the duodenum and the ventral duct drains only a small part into the common bile duct. By embryonic day 16–17 (E16–17) in the mouse the ventral pancreatic bud and the dorsal pancreatic bud fuse and establish communication between the two ducts (Pictet and Rutter, 1972; Sander and German, 1997). At this time the region between the duodenum and the point of fusion undergoes little, or no, enlargement while the pancreatic duct increases in size and forms a highly branched structure that forms the adult pancreatic duct (Jorgensen et al., 2007).

All islet cell types are believed to be derived from one common progenitor cell (Yamaoka and Itakura, 1999) located at the tips of the branching structures (Zhou et al., 2007). In the mouse, endocrine cells are formed continuously from E9.5 until birth, with a major burst in β-cell generation between E13.5 and E16.5 (Ahnfelt-Ronne et al., 2012). Before distinct cell types form, insulin and glucagon, pancreatic polypeptide and amylase mRNA are expressed at embryonic days E9.5, E10.5, and E12.5 respectively (Herrera et al., 1991; Gittes and Rutter, 1992; Deltour et al., 1993). Acini and ductal cells become apparent as histologically distinct structures as early as E15.5 in the mouse; while the islets of Langerhans do not form until around E18.5 (Herrera et al., 1991; Figure 2). After this time, endocrine cell numbers increase rapidly, as a result of ductal epithelial neogenesis rather than multiplication of precursor cells (Deltour et al., 1991; Kaung, 1994). The dorsal primordium is rich in glucagon-containing cells and the ventral primordium is rich in pancreatic polypeptide-containing cells (Malaisse-Lagae et al., 1979; Stefan et al., 1982) the dorsal lobe, derived from the dorsal duct, possess islets with a β-cell core surrounded by α - and δ-cells, while the ventral lobe islets have a mantle of PP and δ-cells surrounding the β-cell core (Tasaka et al., 1989). In the human fetus, endocrine cells begin to bud from the pancreatic duct at around week 10 of gestation. The functional endocrine pancreas continues to develop throughout pregnancy followed by a phase of islet remodeling from late gestation onwards for at least 4 years (Fowden and Hill, 2001; McMillen and Robinson, 2005). Hence, irrespective of species, throughout the process of embryogenesis not all pancreatic islets have the same endocrine cell content.

### Transcription factors controling pancreatic development

**Figure 3:**
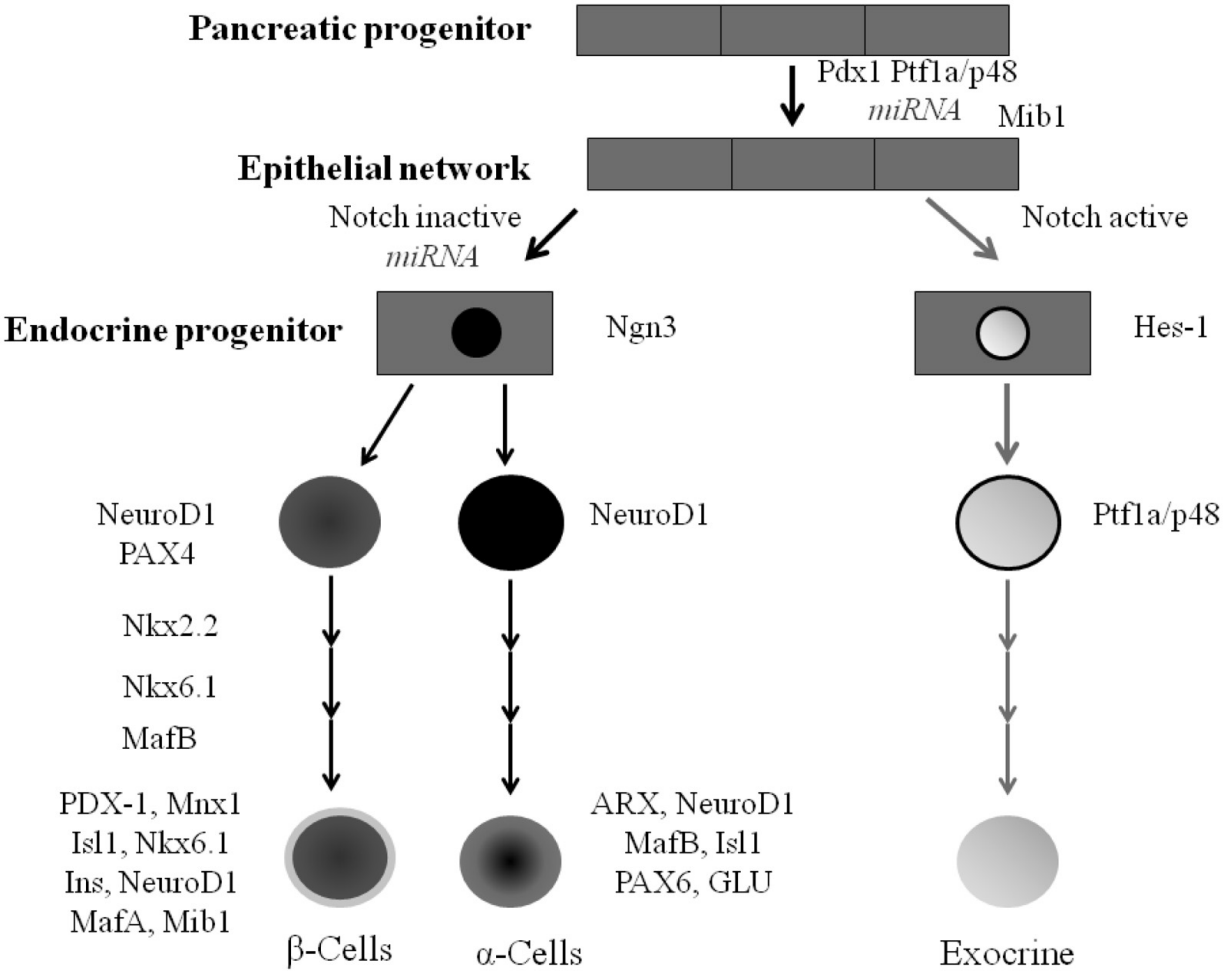
**Pancreatic cell lineage and the role of transcription factors during islet cell type development.** All mature pancreatic cell types are derived from progenitors that express *Pdx-1* and/or *Ptf1a*. *Mnx-1 and Mib-1* act to regulate notch ligand activity. *Ngn3* expression in pancreatic progenitor cells gives rise to ductal, acini, and endocrine progenitor cells. *Nkx2.2* and *Nkx6.1* work alongside other transcription factors including *PDX-1, MafA Mnx1, Isl1* and *NeuroD1* to regulate the formation of the majority of islet β-cells.

The embryological development of the pancreas and the endocrine and exocrine cells is tightly controlled. Throughout these stages of development a network of transcription factors regulate genes that direct cellular differentiation and cell fate (Figure 3). While some transcription factors are only required at specific stages of the formation of the islet cell others are required in multiple stages of endocrine cell development (Murtaugh, 2007).

#### Transcription factors

***Pdx-1.*** Pancreatic duodenal homeobox 1 (Pdx-1) is one of the first transcription factors expressed, with gene expression starting as early as E8.5 in the mouse in the foregut endoderm (Offield et al., 1996). All the cells derived from the endoderm have been shown to express Pdx-1 (Gu et al., 2002). Both the ventral and dorsal pancreatic buds express *Pdx-1* at E9.5 (Offield et al., 1996). At approximately E10 expression of *Pdx-1* mRNA is then downregulated with expression becoming restricted only to endocrine cells in the pancreas and this is maintained in adult β-cells (Ohlsson et al., 1993; Ahlgren et al., 1998). *Pdx-1* itself is an essential mediator of mesenchymal signaling, necessary for the branching morphogenesis involved in ductal network formation of the pancreas at E10.5 (Ahlgren et al., 1996). Germline knockout studies have shown that while knockdown of *Pdx-1* prior to E10.5 has no effect on pancreatic developmental processes (Wescott et al., 2009), the targeted pancreatic deletion at E10.5 or later results in pancreatic agenesis (Ahlgren et al., 1996; Offield et al., 1996). *Pdx-1* contains three principal transcription initiation sites (Sharma et al., 1996) and in the β-cell each of these sites may be activated by the binding of a specific set of transcription factors (Melloul et al., 2002); FOXA2, HNF6, PTF1a, MNX-1, MAFA, HNF, SP1/3, USF1/2 and PDX-1 itself (Harrison et al., 1999; Melloul et al., 2002; Jacquemin et al., 2003; Gao et al., 2008; Vanhoose et al., 2008).

Interestingly, reduced expression of the *PDX-1* gene within the human pancreas has been linked with type 2 diabetes, a rare autosomal dominant form of type 2 diabetes called maturity onset diabetes of the young (MODY4) and pancreatic agenesis (Lin and Vuguin, 2012).

***Ptf1a/p48***. Ptf1a (pancreas specific transcription factor 1a/p48) is a basic helix-loop-helix (bHLH) transcription factor (Beres et al., 2006). It was first identified as a subunit of the trimeric PTF1 transcription factor complex, and has been found to be crucial for the regulation of exocrine gene transcription (Ohlsson et al., 1993; Krapp et al., 1996). Endodermal expression of *Ptf1a/p48* is pancreas specific throughout development; being expressed in endocrine, exocrine and ductal cell types (Kawaguchi et al., 2002). *PTF1a* protein has been detected as early as E8–8.75 in the ventral and dorsal pancreatic ducts (Hald et al., 2008) but by E13.5 expression becomes restricted to acinar precursor cells (Kawaguchi et al., 2002). In adult rodents, PTF1a/p48 transcription factor protein is expressed in acinar tissue and induces amylase and elastase gene expression.

While a deficiency in PTF1a/p48 protein does not inhibit the initial formation of the pancreas it does cause a complete lack of acinar cell development (Krapp et al., 1998; Kawaguchi et al., 2002). Cell lineage studies have shown that this is through cells adopting an intestinal fate rather than becoming cells within the ventral pancreas (Kawaguchi et al., 2002). Defects in the human PTF1a protein are associated with permanent neonatal diabetes mellitus (Masui et al., 2007). It has recently been suggested that *Ptf1a*-mediated control of Delta-like ligand 1 (*Dll1*) expression is crucial for Notch-mediated control of early pancreas development (Ahnfelt-Ronne et al., 2012). The *Ptf1a* activation of *Dll1* within multipotent progenitor cells (MPC) stimulates proliferation and pancreas growth by maintenance of HES1 (hairy and enhancer of split 1) expression and PTF1a protein levels (Ahnfelt-Ronne et al., 2012).

#### Endocrine lineage specification

Differentiation of the cells into each endocrine cell type found within the islet of Langerhans begins at specific time points during embryogenesis. For α-cells this is E9.5, for β-cells E10.5, for δ-cells at E14.5 and lastly PP cells at E18.5. The critical window of differentiation of endocrine cells in humans is from weeks 7 to 23 of gestation (Lin and Vuguin, 2012). Glucagon-producing α-cells are found at week 7, followed by insulin-producing β-cells, somatostatin-producing δ-cells and pancreatic polypeptide PP cells at weeks 8–10 gestation (Lin and Vuguin, 2012). Importantly, the processes that control the development of each cell type are governed by a complex signaling cascade.

***Notch cell signaling***. The notch cell signaling pathway is a well-conserved signaling pathway that regulates cell fate determination during embryonic development as well as the maintenance of tissue homeostasis during adult life (Bigas and Espinosa, 2012). During development Notch signaling must be regulated to balance the expansion of the progenitor pool with the differentiation of mature cell types (Greenwood et al., 2007), with genetic perturbations of this pathway resulting in a change in pancreatic cell fate (Apelqvist et al., 1999). Notch-mediated signaling ensures the appropriate maintenance of the progenitor cell population through the activation of the bHLH factor *Hes1* (Jensen et al., 2000). Loss-of-function experiments have shown that Notch signaling promotes pancreatic progenitor cell self-renewal and/or exocrine lineage commitment (Murtaugh et al., 2003). A reduction in Notch signaling increases the expression of the pro-endocrine gene *Ngn3*, promoting an endocrine fate; while those cells exposed to Notch signaling express *Hes-1* and *p48* mRNA and adopt an exocrine fate (Apelqvist et al., 1999).

***Ngn3***. Neurogenin 3 (NGN3) is one of the most important transcription factors for endocrine development. **A**ll endocrine cell types are derived from progenitor cells expressing the *Ngn3* gene (Gu et al., 2002). The bHLH transcription factor is expressed in two distinct waves (Villasenor et al., 2008). The first phase of *Ngn3* expression begins in murine pancreatic progenitor cells at E8.5–9 resulting in glucagon producing cells, while the second begins at E12 with expression peaking at E15.5 corresponding with endocrine cell differentiation (Gradwohl et al., 2000; Schwitzgebel et al., 2000). *Ngn3* null animals are born without islets, and lack all endocrine cell types, develop type 1 diabetes and die 1–3 days after birth (Gradwohl et al., 2000). *Ngn3* has been associated with several downstream factors involved in endocrine differentiation and cell subtype specification and maintenance, including *Nkx2.2, Nkx6.1, Pax4 Isl1, b2/NeuroD1, Pax6* and *Pdx-1* (Huang et al., 2000; Smith et al., 2003; Collombat et al., 2005).

***Pax4—Arx***. The paired box containing gene 4 *(Pax4)* mRNA is first detected at E9.5 and is transiently expressed in all endocrine progenitors during pancreatic development, being downregulated shortly after birth (Lin and Vuguin, 2012). *Pax4* is expressed downstream of *Ngn3*, with expression being lost in *Ngn3* null mice but not vice versa (Gradwohl et al., 2000; Wang et al., 2004). *Pax4* activity appears essential for the appropriate initiation of β-cell differentiation (Collombat et al., 2005). In the absence of *Pax4*, β - and δ-cells fail to develop and more α - cells are observed (Collombat et al., 2003, 2005; Sosa-Pineda, 2004). The loss of *Pax4* prevents the expression of *Pdx-1, HB9* and insulin mRNA in β-cell precursors (Wang et al., 2004). PAX4 has an opposing action to the transcription factor aristaless-related homeobox gene (ARX) (Collombat et al., 2003). *Arx* gene expression begins at E9.5 during mouse pancreatic development and persists into mature α-cells (Collombat et al., 2003). *Arx* null mice lack α-cells despite having no changes in total islet cell number (Collombat et al., 2005). Therefore, it is the balance of *Pax4* and *Arx* that is crucial for cell fate determination of both α - and β - cells within the islet (Collombat et al., 2005).

***Nkx***. Members of the NK homeodomain transcription factor family are critical regulators of organ development (Stanfel et al., 2005). Of these, *Nkx2.2* and *Nkx6.1* are involved in endocrine cell lineage and are likely to be the most important during pancreatic development.

*Nkx2.2* NKX2.2 is able to bind to both the insulin and *Pax4* promoters (Cissell et al., 2003). The *Nkx2.2* gene is initially dorsally expressed with *Pdx-1* at E8.75 and ventrally at E9.5 (Jorgensen et al., 2007). However, by E15.5 its expression becomes restricted to only endocrine cells (Sussel et al., 1998). *Nkx2.2* deficiency results in a reduction of α - and PP-cells, and the complete loss of β-cells (Sussel et al., 1998) and in the absence of *Nkx2.2*, β-cells fail to activate insulin gene transcription and lack *Nkx6.1*, suggesting that *Nkx2.2* is essential for the specification of the mature β-cell phenotype (Sussel et al., 1998).

*Nkx6.1* Nkx6.1 pancreatic gene expression is similar to that of *Nkx2.2*. In mouse models *Nkx6.1* is expressed in the prospective ventral pancreas domain at E8.75. By E9.0 *Nkx6.1* expression switches from the ventral domain to the dorsal domain until E10.5, when ventral expression of *Nkx6.1* reappears (Jorgensen et al., 2007) and then by E11.5, its expression becomes restricted to the central epithelium (Jorgensen et al., 2007). In adult murine islets, *Nkx6.1* expression is restricted to β-cells (Jensen et al., 1996) where it suppresses glucagon expression and modulates glucose stimulated insulin secretion (Schisler et al., 2005). *Nkx6.1* acts downstream of *Nkx2.2* (Sussel et al., 1998) Not only do *Nkx2.2* single mutant animals lack expression of *Nkx6.1* within the β-cell (Sussel et al., 1998) but the *Nkx6.1* promoter contains a conserved binding site for *Nkx2.2* (Sander et al., 2000). In fact, *Nkx6.1* deficiency does not affect early pancreas development, but it does result in compromised β-cell development (Sander et al., 2000) and mutant animals lacking both *Nkx2.2/Nkx6.1* have a phenotype identical to that of *Nkx2.2* knockout animals (Sander et al., 2000).

#### Maintenance of β-cell identity

***NeuroD1/Beta2***. *NeuroD1/Beta2*, a bHLH, is first expressed in mouse pancreatic cells from E9.5 (Naya et al., 1997). By E12.5, *NeuroD1/Beta2* mRNA is found in both dorsal and ventral pancreatic buds and from E17.5 gene and protein expression is restricted to small clusters of endocrine cells and is rarely detected in ductal epithelial cells (Chu et al., 2001). In adult islets, *NeuroD1/Beta2* is expressed in all endocrine cells of the islet, although its function still remains unknown. While it is believed that *NeuroD1/Beta2* plays a role in regulating insulin (Sharma et al., 1999) and glucagon mRNA expression, mice lacking *NeuroD1/Beta2* are still able to produce functional insulin and glucagon (Chu et al., 2001).

***Large Maf transcription factors***. The Maf family of transcription factors belong to the basic leucine-zipper (bZIP) family that have been associated with the regulation of a number of differentiation processes (Hang and Stein, 2011). The Maf family is divided into two groups (small and large) based on their molecular size (Hang and Stein, 2011). The large Maf proteins MAFA, MAFB, c-MAF and NRL contain a transactivation domain in the N-terminal region and are key regulators of cellular differentiation (Yang and Cvekl, 2007; Hang and Stein, 2011). In adult islets, MAFA, MAFB, and c-MAF are expressed unlike NRL (Matsuoka et al., 2003), supporting a role for NRL in islet cell development.

*MafA*. MafA gene expression starts at E13.5 and continues throughout adulthood in the mouse (Matsuoka et al., 2003). *MafA* knockout mice display no changes in pancreas development, but the mice do go on to develop glucose intolerance and type 2 diabetes as a result of reduced β-cell mass and increased β-cell apoptosis (Zhang et al., 2005). Furthermore, MAFA binds to the insulin promoter and through its interaction with PDX-1 and NEUROD1, activates insulin gene transcription (Olbrot et al., 2002; Aramata et al., 2005).

*MafB*. In the adult mouse pancreas *MafB* is expressed within the islet α-cells where it regulates glucagon gene expression (Artner et al., 2006, 2007). Expression of *MafB* mRNA within the mouse pancreas begins as early as E10.5 in glucagon-positive cells, before expression in islet positive cells at E12.5 (Artner et al., 2006). *MafB* becomes progressively restricted to α-cells postnatally through the downregulation of its expression in β-cells in the first 2 weeks after birth and its expression is undetectable in these cells 3 weeks after birth (Artner et al., 2007). MAFB plays a pivotal role in the development and differentiation of α - and β-cells during pancreatogenesis by directly affecting the transcription of specific genes present in α - or β - cells as opposed to genes which are more ubiquitously expressed in other islet cell types (e.g., *Pax6, Isl1* and *NeuroD1*) (Artner et al., 2007).

***Mib-1***. *Mind bomb 1* (*Mib-1*) encodes an E3 ubiquitin ligase essential for Notch ligand activity (Itoh et al., 2003) that is required for correct proximodistal patterning in the developing pancreas as well as in β-cell formation Horn et al., 2012). Endodermal-specific inactivation of *Mib-1* causes the proximal cells of the developing pancreas to adopt a distal fate resulting in a reduction in β-cells (Horn et al., 2012).

***Mnx-1***. The homeodomain transcription factor MNX1 (HB9) plays a key role in pancreatic development and function (Harrison et al., 1999; Li et al., 1999; Dalgin and Prince, 2012). Murine *Mnx-1* gene expression begins at E8 (Lin and Vuguin, 2012). Work in zebrafish suggests that *Mnx-1* may suppress α - cell fate in β-cell precursors (Dalgin and Prince, 2012). Furthermore, *Mnx-1* knockout mice fail to develop the dorsal lobe of the pancreas with the remnant pancreas having smaller islets of Langerhans with reduced β-cell numbers expressing low levels of *Glut2* and *Nkx6.1* (Harrison et al., 1999).

***Lbl-11/Isl-1***. The transcriptional regulator Islet-1 (*Isl-1*) gene is expressed in the developing mouse pancreas at E8–8.5 (Lin and Vuguin, 2012) but is later restricted only to the endocrine cells of adult islets, where it is involved in the maturation, proliferation and survival of the second wave of islet cells (Du et al., 2009). *Isl-1* directly regulates *MafA*, a potent regulator of the insulin gene and β-cell function (Du et al., 2009). *Isl-1* deficient animals fail to develop functional β-cells, and have a reduced ability to expand their endocrine cell mass postnatally and consequently become diabetic (Du et al., 2009). A nonsense mutation of the *ISL-1* gene has been found in a case of human type 1 diabetic patient. Activation of *Isl-1* is regulated in part by the transcriptional co-regulator, LIM domain-binding protein 1 (LBL-1) (Hunter et al., 2013).

#### Other factors

***microRNAs***. microRNAs (miRNAs) are small non-coding RNA molecules (~19–22 nt) that regulate gene expression by binding to complementary sequences in the 3′UTR regions of specific mRNAs (Ambros, 2004). One of the first miRNAs to be identified in the β-cell was miR-375 (Poy et al., 2004). miR-375 is regulated by several transcription factors involved in pancreatic development and function, including HNF6, INSM1, NGN3, NEUROD1, and PDX-1 (Keller et al., 2007), with knockout studies showing that miR-375 is key to pancreatic development (Poy et al., 2009). Since the identification of miR-375, a great number more miRNAs have been implicated in many aspects of pancreatic development including regulating ductal, exocrine, and endocrine development (Lynn et al., 2007). For example miR-18a plays a role in regulating pancreatic progenitors and exocrine cells through the repression of *Ptf1a* expression (Yang et al., 2012).

***Cell cycle regulators***. Regulators of the cell cycle have been relatively understudied during pancreatic development; Of those studied, p27 is a key regulator in establishing β-cell mass; during embryogenesis accumulates in terminally differentiated β-cells where it acts to maintain a quiescent state (Georgia and Bhushan, 2006). Inactivation of p27 during embryogenesis results in an increase in β-cell mass at birth but interestingly does not affect the postnatal expansion ofβ-cell mass (Georgia and Bhushan, 2006).

### Islet growth and development in neonates

The neonatal period is divided into presuckling, suckling and weaning phases. The presuckling period, which represents postnatal days 1–2, describes the transition between fetal life, where the placenta supplies most of the metabolic needs, and extra-uterine life. During this period the islet adapts with alternating feeding and fasting states whilst the total islet mass continues to grow and in fact almost doubles during these 2 days (Freie et al., 1975). Throughout postnatal days 3–14, the suckling period, β-cell mass and insulin secretion adapt to the composition of the maternal diet and to the dam's feeding patterns. Following an initial reduction in growth and proliferation for all islet cell types during postnatal days 3–4, during the suckling phase there is rapid growth from days 4–10 (Kaung, 1994). During this phase the rate of islet neogenesis is higher than the rate of islet growth, resulting in a large number of small islets (Ferrand et al., 1995). Throughout the mid-stages of the suckling phase there is an increase in the concentration of a number of pancreatic hormones, followed by decrease from the end of the suckling through to weaning. Pancreatic insulin levels are at their highest between postnatal days 7–21 and the levels of glucagon and somatostatin increase considerably from birth reaching a peak between days 4 and 7 (Portha et al., 1974). During the weaning phase, postnatal days 15–22, the whole system is honed to produce a fully developed pancreas. At this time, pancreatic weight doubles every 5–6 days until the end of the weaning phase and subsequently the islet density is reduced (Freie et al., 1975).

### Islet growth and development in the adult

There are three main mechanisms involved in the regulation of β-cell mass in adulthood: i) β-cell replication, which leads to an expansion in β-cell number (hyperplasia), ii) β-cell hypertrophy, which leads to an expansion in individual volume and differentiation and iii) β-cell apoptosis. These processes and the underlying mechanisms have been extensively reviewed previously (Bonner-Weir, 2000). Despite no evidence of spontaneous neogenesis occurring in the adult pancreas, it has now been shown that the neogenesis of islets from ductal cells can be activated by exposure to certain peptides (Bonner-Weir, 2000).

## The effects of maternal obesity and a high fat diet on offspring pancreatic development

The developmental programming hypothesis suggests that conditions during key periods of fetal and early neonatal life may impair pancreatic β-cell development and alter health in adulthood (Zambrano et al., 2006). It is well-established in rodent models that intrauterine growth restriction (IUGR), followed by normal or supranormal nutrition after birth can result in impaired growth of β-cells, a reduction of β-cell mass and insulin content (Pinney et al., 2011; Frantz et al., 2012; Martin-Gronert and Ozanne, 2012) and an increased susceptibility to insulin resistance, visceral obesity, type 2 diabetes and other features of the metabolic syndrome in adulthood (Hales and Barker, 1992; Stocker et al., 2005; Sandovici et al., 2011). There are many models used to study IUGR including maternal low protein diet and placental insufficiency models (reviewed, Schwitzgebel et al., 2009; Portha et al., 2011; Tarry-Adkins and Ozanne, 2011), in this review we will focus on the model of maternal obesity.

In the United Kingdom around 50% of women of childbearing age are either overweight with a body mass index (BMI) of 25–29.9 kg/m^2^ or obese (BMI ≥ 30 kg/m^2^) (Thangaratinam et al., 2012). In 2010 5% of the UK maternity population were severely obese (CMACE, 2010). Not only does increased adiposity affect maternal health but there is also increasing evidence that obesity during pregnancy and lactation can have long term effects on the health of the child, increasing susceptibility to type 2 diabetes (McCance et al., 1994; Nelson et al., 2010; Alfaradhi and Ozanne, 2011; Poston, 2012; Rkhzay-Jaf et al., 2012; Yang and Huffman, 2013).

Over the last decade evidence has grown that exposure of rats to palatable and high-saturated-fat diets *in-utero* and/or during lactation also compromises β-cell development and function in neonatal and weanling offspring (Cerf, 2011; Cerf et al., 2012). A high fat diet (HFD) pre-gestation, during gestation or postnatally will have different effects depending on the exposure time to the diet (Dalgin and Prince, 2012). In the majority of studies, HFD were administered to pregnant and/or lactating rats to programme an adverse metabolic phenotype in the offspring (Cerf, 2010). In neonates, exposure to a maternal HFD throughout gestation was observed to have an adverse effect on β-cell development which resulted in hyperglycemia (Cerf et al., 2005). Similarly offspring of dams fed a HFD through gestation alone showed altered neonatal islet morphology with increased α-cell number and size and with an opposite effect on the β-cell (Cerf et al., 2009). In contrast, offspring of obese dams fed an obesogenic diet throughout gestation and lactation were hyperinsulinemic at 3 months of age (Samuelsson et al., 2008). This has been associated with an increase in pancreatic insulin content, increased islet number and increased β-cell mass in early life (Rkhzay-Jaf et al., 2011), which declines with age due to persistent stimulation (Srinivasan et al., 2008). Similar observations were recorded in sheep where, at lambing, reduced offspring β-cell numbers was associated with an increase in β-cell apoptosis (Zhang et al., 2011).

Previous studies conducted in the rat low-protein model of IUGR, resulted in a reduction of PDX-1 protein expression (Park et al., 2008) as well as a reduction in mRNA expression of *Glut2, Hnf4a, Pdx-1, Rfx6, and Ins* (Pinney and Simmons, 2010; Sandovici et al., 2011; Rodriguez-Trejo et al., 2012). Similarly, exposure to a HFD can also alter both the glucose sensing and insulin signaling mechanism of islets. Studies have shown that offspring of obese over-nourished rat dams have a reduction in insulin, glucokinase and GLUT2 protein expression (Kim et al., 1995; Jorns et al., 2002; Cerf et al., 2006), as well as a reduction in PDX-1 protein (Reimer and Ahren, 2002; Cerf et al., 2009). The alterations of these factors by gestational high fat feeding may contribute to the reduction of β-cell number by similar mechanisms to those seen in maternally undernourished models.

While the mechanisms that govern the adaptation of the β-cell to their altered *in-utero* environment are not fully understood it is in part due to epigenetic modifications, including DNA methylation, histone modifications and microRNAs, of which DNA methylation is the most commonly studied. Methyl donors are sourced from the diet and methylation patterns are established during the early development of many organs including the pancreas. Therefore, methyl donor imbalances could alter these epigenetic patterns and increase an individual's susceptibility to metabolic disease in later life (Waterland and Garza, 2002; Sandovici et al., 2011). The many transcription factors that control pancreatic development and endocrine cell fate may be subjected to epigenetic regulation (Avrahami and Kaestner, 2012; Sandovici et al., 2013). Previous studies of the developing pancreas have shown that epigenetic dysregulation brought about by an altered *in-utero* environment increases the long term susceptibility to type 2 diabetes (Simmons, 2007; Thompson et al., 2010). Park *et al* demonstrated that the development of type 2 diabetes following IUGR is in part due to epigenetic changes that result in the silencing of *Pdx-1* gene (Park et al., 2008). Due to the commonalities in the phenotypes produced by the various models of developmental programming (Portha et al., 2011), similar abnormal metabolic intrauterine milieu such as a maternal HFD may also affect the development of the fetus by permanently modifying gene expression through epigenetic mechanisms.

More recently, a study in rodents has shown that a paternal HFD results in impaired glucose tolerance in his female offspring. By adulthood, these female offspring have impaired β-cell function, associated with reduced islet area and the altered expression of 642 pancreatic islet genes many of which have been shown to be involved in calcium, MAPK- and Wnt-signaling pathways, as well as apoptosis and the cell cycle (Ng et al., 2010). Given the fact that maintaining dams on a HFD for a single week during pregnancy can impair β-cell development and function in the weanlings (Cerf et al., 2007) and that a paternal HFD may affect offspring pancreatic islet development, the combined impact of both maternal and paternal diet may produce an exacerbated or different phenotype. Therefore, it is clearly important to understand the potential impact of maternal and paternal obesity on the offspring.

## Conclusion

Recent studies in both animal models and in humans have highlighted the impact of both increased maternal and paternal obesity and over-nutrition on the *in-utero* and postnatal environment and the consequences of developmental programming on an infant's susceptibility to metabolic disorders in later life. It is therefore essential that we understand these programming signals during the key developmental periods of organs such as the pancreas. The identification and a greater understanding of such programming mechanisms will inform the design of successful maternal intervention strategies, removing the risk of altered organ development and rescue the offspring from developing a detrimental phenotype in later life.

### Conflict of interest statement

The authors declare that the research was conducted in the absence of any commercial or financial relationships that could be construed as a potential conflict of interest.
